# Digital Technology and Media Use by Adolescents: Latent Class Analysis

**DOI:** 10.2196/35540

**Published:** 2022-05-04

**Authors:** Megan A Moreno, Kole Binger, Qianqian Zhao, Jens Eickhoff, Matt Minich, Yalda Tehranian Uhls

**Affiliations:** 1 University of Wisconsin-Madison Madison, WI United States; 2 University of California Los Angeles, CA United States

**Keywords:** digital technology, adolescents, latent class analysis, social media, mobile phone

## Abstract

**Background:**

Digital technology and media use is integral to adolescents’ lives and has been associated with both positive and negative health consequences. Previous studies have largely focused on understanding technology behaviors and outcomes within adolescent populations, which can promote assumptions about adolescent technology use as homogeneous. Furthermore, many studies on adolescent technology use have focused on risks and negative outcomes. To better understand adolescent digital technology use, we need new approaches that can assess distinct profiles within study populations and take a balanced approach to understanding the risks and benefits of digital technology use.

**Objective:**

The purpose of this study was to identify profiles of adolescent technology use within a large study population focusing on four evidence-based constructs: technology ownership and use, parental involvement, health outcomes, and well-being indicators.

**Methods:**

Adolescent-parent dyads were recruited for a cross-sectional web-based survey using the Qualtrics (Qualtrics International, Inc) platform and panels. Technology use measures included ownership of devices, social media use frequency, and the Adolescents’ Digital Technology Interactions and Importance scale. Parent involvement measures included household media rules, technology-related parenting practices, parent social media use frequency, and the parent-child relationship. Health outcome measures included physical activity, sleep, problematic internet use, and mental health assessments. Well-being indicators included mental wellness, communication, and empathy. We used latent class analysis (LCA) to identify distinct profile groups across the aforementioned 4 critical constructs.

**Results:**

Among the 3981 adolescent-parent dyads recruited, adolescent participants had a mean age of 15.0 (SD 1.43) years; a total of 46.3% (1842/3981) were female, 67.8% (2701/3981) were White, and 75% (2986/3981) lived in a household with an income above the poverty line. The LCA identified 2 discrete classes. Class 1 was made up of 62.8% (2501/3981) of the participants. Class 1 participants were more likely than Class 2 participants to report family-owned devices, have lower technology importance scores, have household technology rules often centered on content, have positive parent relationships and lower parent social media use, and report better health outcomes and well-being indicators.

**Conclusions:**

Findings from this national cross-sectional survey using LCA led to 2 distinct profile groups of adolescent media use and their association with technology use and parent involvement as well as health and well-being outcomes. The two classes included a larger Class 1 (*Family-Engaged Adolescents*) and a smaller Class 2 (*At-Risk Adolescents*). The findings of this study can inform interventions to reinforce positive technology use and family support.

## Introduction

### Background

Digital technology and media use is integral to adolescents’ lives; adolescents have been labeled digital natives given that they have had exposure to digital technology their entire lives. Previous studies on digital technology and media use have largely focused on assessing behaviors and outcomes within adolescents as a population, which does not allow for an understanding of the heterogeneity of adolescents’ technology use. Few studies have examined specific subgroups to understand the nuances of digital technology and media use across and within adolescent groups [1-3].

Furthermore, many studies on adolescent technology and media use have focused on risk behaviors and negative outcomes. Previous studies have illustrated that digital technology and media use is associated with negative outcomes such as impaired sleep [4-6], decreased physical activity [5,7,8], problematic internet use (PIU) [9-11], and risk of depression [12]. Although several recent review articles have described both the benefits and risks of technology use [13,14], most individual studies take a risk-centered approach [15,16]. Since the COVID-19 pandemic, many teenagers have experienced social isolation as a result of quarantine and remote learning, making digital tools for connection to peers and family even more important.

Critical constructs to consider in the balance of the risks and benefits of technology and media use include factors such as device ownership, frequency of social media use, and the importance of use [17]. Furthermore, other underlying factors in an adolescent’s offline environment may also be critical to consider. Evidence supports that technology use outcomes may also be affected by parental factors such as household rules around media use or the parent-child relationship [18]. To better understand adolescent digital technology and media use, new approaches that can assess distinct profiles within study populations, consider mitigating factors such as technology use and parental involvement, and take a balanced approach to understanding the risks and benefits of digital technology and media use are needed.

### Technology Devices and Media Use Quantity and Quality

Several aspects of adolescent technology use have been studied through previous research. In the area of device ownership, it is understood that US adolescents’ smartphone access is nearly ubiquitous. The 2018 Pew Internet and American Life Project [19] estimated that 95% of US adolescents have their own personal smartphone. These rates increased from 2014-2015, when 73% of adolescents reported personal smartphone access [20]. The age at which teenagers obtain their first phone has also decreased over time, raising concerns that have prompted campaigns to encourage delaying ownership until an adolescent is in the eighth grade [21,22]. Less is known about other device ownership among teenagers. Although approximately 88% of adolescents report access to a desktop or laptop computer at home, it is less clear who owns these devices [19]. Device ownership may be a contributing factor to technology use outcomes. For example, personal ownership of technology may provide more opportunities for frequent use compared with family-owned devices. Personal ownership of devices may also provide access within private spaces such as bedrooms, which are more challenging to regulate by parents [23]. Furthermore, little is known about access to or ownership of newer devices such as virtual reality (VR) headsets and personal assistant devices.

Another area of focus in previous studies is the quantity of adolescents’ digital technology and media use. For adolescents, one way in which quantity of technology use can be measured is via the age at which they first started using technology, such as the age at which a youth acquired a personal smartphone. Earlier initiation to technology use has been associated with maladaptive outcomes such as problematic technology use [24], supporting that quantity of use over time may be an important factor in determining health outcomes. For adolescent populations, quantity of use is commonly measured by self-reporting hours per day spent using technology and media. However, designing research studies to assess quantity of technology use is not without challenges. In particular, self-reporting the quantity of use is subject to recall bias [25,26].

Increasingly, researchers and health care providers are emphasizing that quality of technology use, beyond just quantity of use, may be just as important if not more in understanding links between technology use and health outcomes. Quality of use could include motivations for use, importance of use, or types of web-based interactions. These quality of use concepts are tied to affordances, which describe properties of artifacts that illustrate how they can be used [27,28]. Affordances have been used to tie adolescents’ digital technology use to their developmental milestones [29]. Grounded in an affordance framework, the Adolescents’ Digital Technology Interactions and Importance (ADTI) scale measures the importance of specific technology interactions rather than platform use [17]. Combining quantity and quality measures of technology use may allow for new insights to better understand benefits and risks for adolescents. Thus, a comprehensive approach to understanding adolescent digital technology use patterns could include what devices an adolescent can own and access, the age at which an adolescent obtained their first smartphone, how often they interact with digital technology and media, and how important those technology interactions are to them.

### Parent Involvement and Relationship

The 2016 American Academy of Pediatrics (AAP) media policy statement, *Media Use in School-Aged Children and Adolescents*, emphasized the role of parents in adolescents’ media lives [30]. A key role for parents is to serve as mediators of media use for their children, and studies suggest that parental efforts may have varied impacts on adolescents’ media use [31]. Parents often struggle with setting and enforcing media rules such as removing technology from children’s bedrooms [32,33].

Parents’ own technology experiences may also affect their children. Studies have found that parents have varied attitudes toward technology and varied engagement with technology [34]. Parents who struggle with limiting their own technology use may have challenges in moderating their children’s technology use [35]. For parents who prioritize technology use, these behaviors may be modeled for their children. In fact, families with media-centric parents typically have children who report more media use [18].

Parenting style and parent relationship are additional factors in the balance of risk and beneficial outcomes that adolescents experience. Factors associated with positive media use in families have included positive general family functioning, parental involvement, and open communication styles between parents and adolescents [36]. Thus, although it is important to understand the role of technology use in influencing adolescent health and well-being, it is also critical to better understand how the family context affects adolescent technology-related outcomes.

### Health Outcomes

There are several health concerns that have been associated with digital technology and media use influence and affect. First, technology and media use has been shown to negatively affect sleep by delaying bedtime as well as through exposure to light from screens disrupting melatonin levels [4-6]. Second, decreased physical activity has been associated with the sedentary nature of most media use [5,7,8]. Third, PIU is defined as “Internet use that is risky, excessive or impulsive in nature leading to adverse life consequences, specifically physical, emotional, social or functional impairment” [37]. Studies support that components of PIU include compulsive use and anxiety when not able to access the internet [9].

Adolescents’ mental health has also been a common research topic related to potential negative consequences of media use. Studies have found associations between increased social media use and decreased life satisfaction [38,39], increased risk of depression [38,40], worsened body image and decreased self-esteem [40-42], increased fear of missing out (FOMO) [43], and reduced well-being [44]. These studies suggest that media use may have a negative impact on mental health for adolescents. However, other studies have found that social media use does not affect life satisfaction [45,46] nor depression [47]. Some have argued that data support that media use may negatively affect *some* adolescents but caution that overstating these relationships to apply to adolescents as a whole is not warranted [48].

### Well-being

Studies focusing on ways in which technology influences well-being have found positive associations with increased social support and learning [49,50]. Another study found that adolescents described their affective experiences on social media to include feeling happy and closer to friends, supporting adolescent well-being [50]. Youths may be motivated to adopt digital health technology that includes a social component as it enhances communication skills, enables a sense of belonging and perspective taking and thus increases social support [51,52]. These factors, in turn, may reduce stress or physical illness and improve psychological and physical well-being [53,54].

### Study Purpose

The purpose of this study was to understand patterns across adolescents’ digital technology and media use, including four evidence-based constructs: (1) technology ownership and use, (2) parental involvement, (3) health outcomes, and (4) well-being indicators. Most studies to date have focused on 1 outcome, such as depression, or on a category of outcomes, such as mental health. This study builds on that literature by using the power of latent class analysis (LCA) to examine critical constructs and understand patterns across and within groups. The emerging understanding that adolescents’ media use is not homogeneous and the critical role of parents in moderating youth media use informed this study’s purpose. The purpose of this study was to use LCA to develop profiles representing benefits and risks as well as parental influence associated with digital technology use.

## Methods

### Study Design

A national Qualtrics (Qualtrics International, Inc) cross-sectional web-based research panel was engaged during February-March 2019 to collect data for this LCA study.

### Ethics Approval

The Institutional Review Board at the University of Wisconsin approved this study (2018-0781).

### Setting and Participants

Our goal was to achieve a national sample of youths to complete a web-based survey. Compared with traditional survey approaches such as in-person, phone, or mail recruitment, web-based survey panels offer broader reach and lower costs in data collection [55]. We selected the web-based survey platform Qualtrics for several reasons. First, although web-based survey platforms do not use weighting, previous studies have shown that web-based survey approaches using tools such as Qualtrics can achieve demographic attributes that are typically within a 10% range of their corresponding values in the US population [56]. Second, unlike other platforms such as Mechanical Turk, Qualtrics allows for the recruitment of youths via approaching parents for consent as a first step. Third, there is strong and growing literature around the use of Qualtrics to recruit youth samples in the United States, including studies on media [57,58].

Between February 2019 and March 2019 a Qualtrics survey manager recruited adult panel participants who indicated that they had adolescent children aged 13-18 years who spoke English. Parents who met these criteria were provided with information about the survey and an opportunity to complete informed consent forms for themselves. The informed consent process notified potential participants of the study purpose and research team, of the survey length, that the survey had questions for the parents and adolescents to answer independently, that the survey was voluntary, of the Qualtrics incentive points that would be provided upon completion, and of how the study data would be stored and used. The survey information section stated that the researchers would not request any personal information about the participants. Parents also provided consent for their child’s participation if the child was aged <18 years. Once parental consent was obtained, the parents completed early sections of the survey on the Qualtrics platform. After completing the parent portion, the parents were instructed to pass the device to the adolescent participant. The adolescent was provided with study information and an opportunity to provide assent. Adolescents who provided assent were allowed to begin the survey. Adolescents aged 18 years provided consent and were allowed to begin the survey.

The target population for this study was adolescents aged 13-18 years who were US residents and English-speaking. Using Qualtrics panels of adult participants (a *closed* survey population), we recruited parent-adolescent dyads to allow for parent as well as adolescent input. We established parameters for Qualtrics to recruit a sample consistent with the race and ethnicity representative of the US census population for adults [56]. Qualtrics representatives recruited parents from their panels using emails and SMS text messages. Qualtrics processes ensured that all recruited participants had completed enrollment in Qualtrics panels, and the participants could only complete the survey a single time.

Our sample size estimates were calculated using estimates for LCA [59,60], which supports approximately 1:3-1:4 ratios of number of items to number of participants to achieve a full range of potential number of latent classes with a minimum of 0.8 power. We estimated that approximately 70 items would be included in our LCA process, and we then increased our sample size to allow for investigation of differences in demographic factors such as adolescent age, gender, or race as well as to account for incomplete surveys affecting our final sample size for analysis. Thus, our planned sample size was 4000 parent-adolescent dyads (N=8000 participants).

### Survey Measures

#### Overview

Our goal for this study was to include measures that represented four key constructs: (1) technology ownership and use, (2) parental involvement, (3) health outcomes, and (4) well-being indicators. These 4 constructs were identified based on the categorization of evidence in the literature describing critical and well-established factors associated with adolescent technology use. Our strategy to identify concepts or scales to assess within each construct involved conducting a review of the literature to identify validated scales or measurement tools within each topic area. We cross-referenced those validated scales or measurement tools with existing literature and review articles that described critical concepts in adolescent digital technology and media use. In cases in which a key concept was described repeatedly in the literature but no validated scale existed, we used existing items that had been used in large studies such as the Pew Internet and American Life Project.

For established measurement scales, our goal was to include categorical variables representing high- or low-score values. Thus, for scales with established cutoffs for the summary score, we used these to dichotomize or categorize scores for inclusion in the LCA. For scales without such empirical cutoffs, we dichotomized scores at the median for inclusion in the LCA. In the paragraphs that follow, we describe survey measures and instruments according to the 4 key constructs of focus in this study. In the survey delivered to the participants, the order of measures delivered was randomized. Most survey pages included a single measurement tool or instrument. The participants were allowed to review and change their answers during the course of the survey.

#### Technology Ownership and Use Measures

These survey measures were answered by the adolescent participants. Measures included individual questions about device ownership and age of first smartphone ownership, assessments of frequency of social media use, and the ADTI scale.

##### Personal and Family Device Ownership and Bedroom Access: Adolescent Participants

To assess technology device ownership, we modeled questions after those in previous Pew Internet and American Life surveys to assess device ownership of adolescents and of the family [19,20,61,62]. Furthermore, given that the AAP recommends limiting media device use in bedrooms [30,63], we included questions about which devices were allowed to be used in the adolescent’s bedroom. The participants were asked, “Which of the following devices do you own? Select all that apply”; “Which of the following devices does your family own? Select all that apply”; and “Which of the following do you have access to in your bedroom? Select all that apply.” Response options included television, computer, tablet, video games, smartphones with internet access, cell phones without internet access, VR devices, wearable devices (ie, smartwatches), personal assistants (ie, Alexa), other, and/or none. Each individual response regarding device ownership and bedroom access was included as a bivariate (yes or no) in the LCA.

##### Social Media Use Frequency: Adolescent Participants

Given that social media is a main component of adolescents’ technology use [19], we assessed social media use frequency. Social media use can include two roles: consumer and creator of content. Thus, we asked about the frequency with which the adolescents checked social media and posted on social media modeled after the Pew Internet and American Life Project surveys [19]. Response options included *almost constantly*, *a few times an hour*, *once an hour*, *a few times a day*, *once a day a few times a week*, *once a week*, and *never*. These responses were clustered into three categories: responses representing daily or more use, responses representing weekly but not daily use, and responses indicating less than weekly use.

##### Age of Acquiring a First Personal Smartphone

We asked the adolescents to report the age at which they acquired their first personally owned smartphone with internet access. We categorized the ages as follows: <11 years, 12-14 years, 15-17 years, or not yet having a smartphone of their own.

##### ADTI Scale: Adolescent Participants

Technology interactions and their importance were measured using the ADTI scale, which has been validated in previous work [17]. This scale includes 18 items and 3 factors. For each item, the participants were asked *How important, if at all, is it for you to use media and technology platforms for the following purposes?* The participants responded using a 5-point Likert scale ranging from *not at all important* to *extremely important*. The three subscale factors and an example item for each included (1) technology to bridge online/offline experiences (example item: *look into or follow an event you may attend*), (2) technology to go outside one’s identity or offline environment (example item: *explore your sexuality*), and (3) technology for social connection (example item: *direct message someone*). The Cronbach *α* scores for the three subscales were .87 (factor 1), .90 (factor 2), and .82 (factor 3), and .92 for the total scale. The ADTI scores were included in the LCA as a total score and as 3 individual subscale scores.

#### Parent Involvement

This section of questions included some measures answered by the parents and some answered by the adolescents.

##### Household Technology Rules: Parent Participants

The parent participants were asked how strongly they agreed or disagreed with 7 statements related to the presence of or engagement in household technology rules at home. The statements were modeled after the suggested parent rules and role modeling of the AAP Family Media Use Plan [30]. These rules include three key concepts described in the literature regarding parenting technology behaviors: active mediation (communication), restrictive mediation (limits on time or content), and social co-use [64-66]. These statements were tested in a previous intervention [67]. For each item, the parent participants were asked whether the rule was present in their household. Example statements included *My house had rules about “friending” someone who is unknown off-line* and *My house has rules about viewing screens around bedtime*. The participants were asked to select from a 5-point Likert scale from *strongly agree* to *strongly disagree* for each of the statements. Responses were dichotomized into *agree* or *neutral/disagree* to represent whether the individual rule was or was not present at home for inclusion in the LCA.

##### Parent Social Media Use Frequency: Parent Participants

Similar to the approach used with the adolescent participants, we asked the parents about the frequency with which they checked social media and posted on social media modeled after the Pew Internet and American Life Project surveys [19]. Response options included *almost constantly*, *a few times an hour*, *once an hour*, *a few times a day*, *once a day a few times a week*, *once a week*, and *never*. These responses were clustered into three categories: responses representing daily or more use, responses representing weekly but not daily use, and responses indicating less than weekly use.

##### Internet-Specific Parenting Practices: Adolescent Participants

The adolescent participants were asked to complete the internet-specific parenting practices scale, which describes practices that their parents use to moderate their children’s use at home [68]. This 12-item scale has 3 subscales. One subscale assesses rules regarding time on the web and has response options of never=1, rarely=2, sometimes=3, often=4, and very often=5. The second subscale measures rules regarding content of internet use, and the third subscale assesses quality of communication regarding internet use. An example item from the third subscale is *When my parents/guardians and I talk about my internet use I feel I’m taken seriously*. The latter 2 subscales have response options using a 5-point Likert scale from *absolutely not true* to *absolutely true*. Responses for each subscale were dichotomized into high- or low-score categories based on the median for inclusion in the LCA published in the literature. The median score for subscale 1 (time) was 18, the median score for subscale 2 (rules) was 12, and the median score for subscale 3 (communication) was 12. This scale had good internal consistency, with a Cronbach *α* of .85.

##### Parent-Adolescent Relationship: Adolescent Participants

The adolescent participants completed the Parent-Adolescent Relationship Scale to assess the quality of the participants’ relationship with their parents who also participated in this study [68]. This validated scale includes 8 items and 2 subscales. The first subscale measures the participant’s identification with their parents and includes items such as *She/He is a person I want to be like*. Responses for this subscale use a 5-point Likert scale from *strongly disagree* to *strongly agree*. The second subscale assesses perceived parental supportiveness and includes items such as *How often does she/he praise you for doing well?* Responses use a 5-point Likert scale of *Never* to *Always*. The internal consistency of this scale was good as indicated by a Cronbach *α* between .72 and .74 with mothers and of .82 with fathers. This measurement was dichotomized and included in the LCA as high and low parent-adolescent relationship based on the published cutoff of >24 indicating a high-quality parent-adolescent relationship.

#### Digital Technology and Media-Related Health Outcomes

##### Overview

These survey items were all answered by the adolescent participants. These measures included a comprehensive set of physical and mental health concerns noted in the literature as being associated with digital technology and media use among adolescents. These measures included physical health outcomes, such as sleep and physical activity, as well as mental health outcomes, including PIU, depression, anxiety, FOMO, and body image concerns.

##### Physical Activity: Adolescent Participants

Physical activity was evaluated using the physical activity scale. This scale included 3 items assessing the frequency with which the adolescents participated in sports and exercised outside school [69]. For each item, the participants responded from *never* to *4 times or more a week*. The Cronbach *α* for this scale is reported by age, including .69 for 13 years and .74 for 15 years. Responses were dichotomized as high or low physical activity using the median reported physical activity (9) for inclusion in the LCA.

##### Sleep: Adolescent Participants

Sleep was assessed using the validated Pediatric Daytime Sleepiness Scale [70]. This 8-item scale includes items such as *How often do you fall asleep or get drowsy during class periods?* and *How often do you fall back to sleep after being awakened in the morning?* Response options include *never* *seldom*, *sometimes*, *frequently*, and *always*. The Cronbach *α* for this scale was .81. Responses were dichotomized as high or low level of sleepiness using the median reported level of sleepiness (13) for inclusion in the LCA.

##### PIU: Adolescent Participants

PIU was measured using the validated 3-item Problematic and Risky Internet Use Screening Scale survey [71]. The participants responded to 3 questions related to internet use, including *how often do you experience increased social anxiety due to your internet use*, using a Likert scale. Response options included never=1, rarely=2, sometimes=3, often=4, and very often=5. The Cronbach *α* for this scale was .96. A total score of ≥3 indicated a risk for PIU, and responses were dichotomized as at risk or not at risk for the LCA.

##### Depression: Adolescent Participants

Depression was measured using the 9-item Patient Health Questionnaire (PHQ-9) [72]. This 9-item scale asks participants how often they have experienced the following symptoms in the past 2 weeks. Example items include *little interest or pleasure in doing things* and *feeling down, depressed, or hopeless*. Response options use a 4-point Likert scale from *not at all* to *nearly every day*. The PHQ-9 had a Cronbach *α* of .82 [73]. We used the validated categorization of no depression (scores 0-4), minimal depression (scores 5-9), mild depression (scores 10-14), moderate to severe depression (scores 15-19), and severe depression (scores >20) in the LCA.

##### Anxiety: Adolescent Participants

Anxiety was measured using a validated reduced version of the Screen for Child Anxiety-Related Emotional Disorders scale [74]. This 5-item scale asks participants how true each of the items is for that participant, including statements such as *People tell me I worry too much*, *I am scared to go to school*, and *I am shy*. Response options include not true or hardly ever true=0, sometimes true=1, and true or often true=2. The Cronbach *α* has been reported as .70 to .90. We used the cutoff score of 3 to categorize the participants as at risk or not at risk for inclusion of scores in the LCA.

##### FOMO: Adolescent Participants

FOMO was measured using the Fear of Missing Out scale [75]. This scale includes 10 items that measure FOMO, or the fear that others are having more rewarding experiences that participants were absent from or missed. Example items include *I fear my friends have more rewarding experiences than me*, *I get anxious when I don’t know what my friends are up to*, and *When I miss out on a planned get-together it bothers me*. Response options include a 5-point Likert scale from *Not at all true of me* to *Extremely true of me*. The FOMO scale had good internal consistency (Cronbach *α*=.90). We dichotomized responses as high FOMO and low FOMO based on the median summary score (23) for the LCA.

##### Body Image: Adolescent Participants

Body image was measured using the Body Image Scale [76]. This scale measures participants’ general satisfaction or dissatisfaction with their body and appearance. Example items include *By and large, I am satisfied with my looks* and *I would like to change a good deal about my body*. Items were rated with response options of 1=does not apply at all, 2=does not apply well, 3=applies somewhat, 4=applies fairly well, 5=applies well, and 6=applies exactly. The Cronbach *α* for this scale was .82, indicating good internal consistency. We categorized responses as low body image and high body image based on the median summary score of 17 for the LCA.

#### Well-being Indicators: Adolescent Participants

These measures were answered by the adolescent participants. Well-being indicators included the Mental Well-being scale [77], the Interpersonal Reactivity Index to measure empathy and perspective taking [78], a communication scale [79], the Comprehensive Inventory of Thriving (CIT) [80], and an assessment of extracurricular activities.

##### Mental Well-being Scale: Adolescent Participants

Mental well-being was measured using the Short Warwick-Edinburgh Mental Well-being Scale [77]. This 7-item validated scale asks participants to indicate how often they agreed with the statement over the past 2 weeks. Example items include *I’ve been feeling optimistic about the future*, *I’ve been feeling relaxed*, and *I’ve been thinking clearly*. Response options include a 5-point Likert scale from *none of the time* to *all of the time*. The internal consistency reliability of the Short Warwick-Edinburgh Mental Well-being Scale was strong (Pearson Separation Index=0.84). We dichotomized the summary scores by the median (27) for inclusion in the LCA.

##### Interpersonal Reactivity Index: Adolescent Participants

The Interpersonal Reactivity Scale was included, which has subscales to measure empathy and perspective taking [78]. This scale includes 14 items, 7 of which measure perspective taking, or the tendency to spontaneously adapt the psychological point of view of others. Items such as *I try to look at everybody’s side of a disagreement before I make a decision* were included. The subsequent 7 items assess empathetic concern, or *other-oriented* feelings of sympathy and concern for unfortunate others. Items such as *I am often quite touched by things that I see happen* and *I often have tender, concerned feelings for people less fortunate than me* were included in the empathetic concern subscale. Response options use a 5-point Likert scale ranging from *Does not describe me well* to *Describes me very well*. The internal reliability of the scale was good (Cronbach *α*=.71-.77). These 2 subscales were each dichotomized at the median values: 16 for perspective taking and 18 for empathetic concern.

##### Communication Skills: Adolescent Participants

The Communication Skills Scale was used to measure communication skills [79]. This validated scale includes 23 items that assess the effectiveness of one’s communication skills, with items such as *When talking to someone, I try to maintain eye contact* and *I try to see the other person’s point of view*. Responses use a 5-point Likert scale from *Never* to *Always*. The internal reliability of this scale was good (Cronbach *α*=.83). Summary scores were dichotomized as high and low communication skills based on the median summary score (52) to categorize for inclusion in the LCA [81].

##### CIT (Support, Learning, and Loneliness): Adolescent Participants

The CIT was used with the subscales of support, learning, and loneliness [80]. This validated scale includes 9 items and 3 factors. For each item, the participants were asked to indicate their agreement with the statements. Example items for support include *There are people I can depend on to help me*, example items for learning include *Learning new things is important to me*, and example items for loneliness include *I often feel left out*. Responses use a 5-point Likert scale from *strongly disagree* to *strongly agree*. The Cronbach *α* for these subscales was .88 for support, .84 for learning, and .90 for loneliness. We dichotomized the responses using the median score for each of the three subscales for inclusion in the LCA: the median for support was 14, the median for learning was 12, and the median for loneliness was 6.

##### Extracurricular Activities: Adolescent Participants

We assessed involvement in extracurricular activities via the Involvement in Extracurricular Activities measure [82]. This 4-item scale asked the participants how many hours they spent during an average week on each statement. Example items include *...in clubs or organizations at school (other than sports)* and *...in clubs or organizations outside of school*. Response options include 0 hours, 1-2 hours, 3-5 hours, 6-10 hours, and ≥11 hours. This scale is described as being intended to describe diverse activities; thus, the validation paper recommends against a Cronbach *α* [82]. This measurement was dichotomized as high or low extracurricular activity time based on the median summary score of 8 for inclusion in the LCA.

#### Demographic Variables: Adolescent and Parent Participants

Demographic variables reported by the adolescent participants included self-reported age, which was dichotomized to represent older adolescents (aged 16-18 years) and younger adolescents (aged 13-15 years). The adolescents reported their gender identity, which was categorized as female identity (female sex and transgender females), male identity (male sex and transgender males), and nonbinary and other identities. The adolescents described their ethnicity as Hispanic or Latino or non-Hispanic or Latino. The adolescents selected all categories that described their race using the US census categories to include White, Black or African American, Asian, American Indian, Native Hawaiian, or Pacific Islander, multiracial, and other. On the basis of a previous study showing that religion mitigated what teenagers posted, religious identity was asked about and dichotomized into reporting a religion or not [83].

We asked the parent participants to report their annual household income. This variable was dichotomized using the US census data that defined poverty such that the participants were categorized as above or below the poverty line [84]. Parent demographics also included gender and marital status.

### Analysis

#### Overview

LCA is a nonparametric statistical method that characterizes otherwise unobservable groups based on individuals’ response patterns to multiple observable variables [85]. Thus, distinctive mutually exclusive subgroups within a population can be empirically identified using LCA [86]. Specifically, we used LCA to identify distinct participant profile groups of multifaceted constructs; in this study, these constructs included technology ownership and use, parental involvement, health outcomes, and well-being indicators. We included measures representing these constructs as well as items representing demographics as variables in the LCA.

#### LCA Data Preparation

Some measures were included in the LCA as individual items, such as demographic variables and individual digital device ownership items. Other measures included in the LCA were summary scores derived from validated scales, such as the PHQ-9 to assess depression. To address missing data, we used the following process. For measures that consisted of multiple items toward a summary score, if >70% of items were present, we rescaled the total score based on the available items [87].

To prioritize the identification of subgroups representing distinct profiles as our outcome, we used all measurement scale outcomes as bivariate or categorical inputs for analysis. For scale scores with published cutoffs, we used those scores to create input categories. In cases in which there were no published score cutoffs, we used the median value to separate scores into high and low and included the high or low designations as inputs in the LCA. For demographic data on a continuous scale, we used the median as a cutoff to distribute the variables into 2 categories.

#### LCA Procedure

We planned to include items in the LCA with both relevance and frequency for our study purpose in our data set. We began our analysis procedures with all 68 items that were selected a priori for the survey. The Lo-Mendell-Rubin [88] likelihood ratio test was used to identify the number of classes. Specifically, the likelihood function of the LCA model with *k* classes was compared with the likelihood function of an LCA model with *k* – 1 classes. *P*<.05 indicated that the model with *k* classes provided a better ﬁt than the model with *k* – 1 classes.

After identification of an initial 2-class model, we used the Fisher exact test to compare items between classes for the preliminary model. Items with *P*<.10 were reviewed. Some items with *P*<.10 were identified as subitems from within larger concepts; thus, we retained those within the model. The final model included all 65 items. The statistical analyses were conducted using SAS software (version 9.4; SAS Institute Inc) and M-Plus software (version 8; Muthen & Muthen 1998-2017).

## Results

### Participants

A total of 4592 adolescent-parent dyads began the survey, of which 3981 (86.7%) completed the survey. Participants were excluded for not completing ≥75% of the survey, responding with single-response selections across multiple survey measures, and 13.3% (611/4592) of adolescent-parent dyads were excluded for these reasons. Regarding the included participants, the adolescents had a mean age of 15.0 (SD 1.43) years, 46.3% (1842/3981) were female, 67.8% (2701/3981) were White, and 75% (2986/3981) lived in a household with an income above the poverty line (Table 1).

**Table 1:** Demographic information of the participants (N=3981).

Variable and categories				Values
**Adolescent demographic information**				
	**Adolescent age (years), n (%)**			
		12-14	1589 (39.9)	
		15-18	2376 (59.7)	
	Adolescent age (years), mean (SD)		15.02 (1.43)	
	**Adolescent sex, n (%)**			
		Female (cisgender and transgender)	1842 (46.3)	
		Male (cisgender and transgender)	2081 (52.3)	
		Other (nonbinary)	58 (1.5)	
	**Adolescent race, n (%)**			
		White	2701 (67.8)	
		Black and African American	586 (14.7)	
		Native American and Alaskan Indian	137 (3.4)	
		Asian	197 (4.9)	
		Multiracial	178 (4.5)	
		Other	182 (4.6)	
	**Adolescent ethnicity, n (%)**			
		Non-Hispanic	3222 (80.9)	
		Hispanic	705 (17.7)	
	**Adolescent identifies with a religion, n (%)**			
		Yes	2688 (67.5)	
		No	1293 (32.5)	
**Parent demographic information, n (%)**				
	**Household income below poverty line**			
		No	2986 (75)	
		Yes	975 (24.5)	
	**Parent sex**			
		Female (cisgender and transgender)	2672 (67.1)	
		Male (cisgender and transgender)	1903 (47.8)	
		Other (nonbinary)	17 (0.4)	
	**Parent relationship status**			
		Married or partner	2902 (72.9)	
		Not married or partner	1047 (26.3)	

### LCA Findings

#### Overview and Class Structure

The LCA revealed two distinct classes to describe our four areas of focus for this study: (1) technology ownership and use, (2) parental involvement, (3) health outcomes, and (4) well-being indicators. Class 1 represented approximately two-thirds of the participants (2501/3981, 62.8%), and Class 2 represented approximately one-third of the participants (1480/3981, 37.2%).

Regarding demographic variables, Class 1 tended to be slightly older and identify as female compared with Class 2, which had more male and nonbinary participants. There were some statistically significant differences between the 2 classes in terms of ethnicity and race. Class 1 participants more often described themselves as non-Hispanic, Black, multiracial, or other races compared with Class 2 participants. In contrast, Class 2 participants often described themselves as Hispanic, Asian, or Native American. Class 1 participants were more likely to be religious and live below the poverty line compared with Class 2 participants. Table 2 presents demographic comparisons between the 2 classes.

**Table 2:** Distribution of demographic variables included in the latent class analysis in the 2-class model (N=3981).

Variable			Class 1 (n=2501; %)		Class 2 (n=1480; %)		*P* value^a^
**Age (years)**							.06
	13-14	38.9		*41.9* ^b^			
	15-18	*61.1*		58.02			
**Sex**							<.001
	Female	*48.3*		42.8			
	Male	*51.2*		*54.05*			
	Other	*0.48*		*3.11*			
**Ethnicity**							<.001
	Hispanic and Latino	16.1		*21.11*			
	Non-Hispanic and non-Latino	*83.92*		78.9			
**Race**							.002
	White	67.7		*68.04*			
	Black or African American	*15.35*		13.6			
	Asian, Asian Indian, or other Asian	2.9		*4.39*			
	American Indian, Native Hawaiian, or other Pacific Islander	4.2		*6.2*			
	Multiracial	*4.9*		3.7			
	Other	*4.9*		4.1			
**Religion**							.02
	Religious	*33.8*		30.3			
	Nonreligious	66.21		*69.7*			
**Parent relationship status**							.08
	With a partner	72.54		*75.1*			
	Not with a partner	*27.5*		24.9			
**Household socioeconomic status**							.07
	Above poverty line	74.43		*77*			
	At or below poverty line	*25.6*		22.99			

^a^*P* value from chi-square test.

^b^Italicization denotes the class with the majority percentage for each measure.

#### Technology Ownership and Use

Regarding media use variables, Class 1 participants were more likely to report *family ownership of technology devices* for most devices, including computers, tablets, video game consoles, televisions, and smartphones. Class 2 participants were more likely to report that they, as adolescents, owned *personal technology devices*, including televisions, computers, tablets, and video games, compared with Class 2 participants. Furthermore, Class 2 participants were more likely to report both family and individual ownership of newer devices such as VR headsets, wearable devices, and personal assistants. Class 2 participants were also more likely to report access to their devices in their bedrooms compared with Class 1 participants.

Regarding age at which the first smartphone was acquired, Class 1 participants were more likely to report acquiring their first smartphone between the ages of 12 and 14 years as well as being more likely to report not having a smartphone compared with Class 2 participants. Class 2 participants were more likely to report acquiring their first smartphone either early (before the age of 11 years) or later (ages 15-17 years). Furthermore, Class 2 was also more likely to report both checking and posting on social media daily compared with Class 1.

Assessing the importance of technology interactions using the ADTI, Class 1 consistently had lower scores and, in some cases, only half of the summary scores for all ADTI subscales compared with Class 2. Table 3 presents these findings.

**Table 3:** Distribution of technology behaviors included in the latent class analysis in the 2-class model (N=3981).

Variable			Class 1 (n=2501; %)		Class 2 (n=1480; %)		*P* value	
**Family device ownership**								
	Television	*98.4* ^a^		89.1		<.001		
	Computer	*85.9*		78.8		<.001		
	Tablet	*74.9*		68		<.001		
	Video game console	*80.8*		72.2		<.001		
	Smartphone with internet access	*94.8*		79.4		<.001		
	VR^b^ devices (such as Samsung Gear VR and Oculus)	15.9		*28.1*		<.001		
	Wearable devices (such as smartwatches and fitness trackers)	*34.4*		32.2		.15		
	Personal assistants (such as Alexa and Google Home)	33.9		*34.3*		.78		
**Adolescent device ownership**								
	Television	57.2		*63.4*		<.001		
	Computer	40.7		*54.5*		<.001		
	Tablet	41.3		*46.8*		<.001		
	Video game console	48.5		*53.1*		.005		
	Smartphone with internet access	*83.2*		69.7		<.001		
	VR devices (such as Samsung Gear VR and Oculus)	6.8		*18.5*		<.001		
	Wearable devices (such as smartwatches and fitness trackers)	13.4		*18.2*		<.001		
	Personal assistants (such as Alexa and Google Home)	8.9		*11.8*		.003		
**Adolescent device access in bedroom**								
	Television	*71.8*		69.2		.08		
	Computer	37.9		*52.4*		<.001		
	Tablet	38.9		*45.5*		<.001		
	Video game console	42.3		*51.1*		<.001		
	Smartphone with internet access	*76.3*		66.4		<.001		
	VR devices (such as Samsung Gear VR and Oculus)	5.7		*16.5*		<.001		
	Wearable devices (such as smartwatches and fitness trackers)	11.6		*16.5*		<.001		
	Personal assistants (such as Alexa and Google Home)	8.7		*12.1*		<.001		
**Age of first smartphone (years)**								<.001
	<11	27.7		*34.4*				
	12-14	*56.8*		51.2				
	15-17	10.3		*12.5*				
	Does not own a smartphone	*5.2*		1.9				
**Adolescent social media checking frequency**								<.001
	Once a day or more	71.8		*87.7*				
	Once a week or more	*15.5*		9.3				
	Less than once a week or never	*12.8*		2.9				
**Adolescent social media posting frequency**								<.001
	Once a day or more	48.4		*81.9*				
	Once a week or more	*22.1*		10.9				
	Less than once a week or never	*29.5*		7.2				
**Adolescent importance of technology interactions**								<.001
	Subscale 1: technology to bridge online/offline experiences and preferences	37.4		*71.8*				
	Subscale 2: technology to assist in going beyond one’s current identity, mood, or environment	33.2		*84.5*				
	Subscale 3: technology for social connection	42.8		*67.7*				

^a^Italicization denotes the class with the majority percentage for each measure.

^b^VR: virtual reality.

#### Parent Involvement

Regarding parent involvement, Class 1 parent participants were more likely to report all categories of household rules compared with Class 2 parent participants. Class 2 parent participants were more likely to report no household rules or boundaries around technology use. Parents in Class 2 were also more likely to report more than daily social media checking and posting compared with parents in Class 1.

Regarding internet-specific parenting styles, adolescents in Class 1 were more likely to report strict rules around internet content and positive parental communication about media use. In comparison, adolescents in Class 2 were more likely to report strict internet rules around time spent on technology and were less likely to experience high-quality communication with their parents about technology. Table 4 illustrates the findings on parent involvement and rules previously described. Finally, adolescents in Class 1 were more likely to report a higher-quality parent relationship compared with adolescents in Class 2.

**Table 4:** Distribution of parent involvement and rules included in the latent class analysis in the 2-class model (N=3981).

Variable		Class 1 (n=2501; %)	Class 2 (n=1480; %)	*P* value	
**Household media rules: parent-reported**					
	My house has no rules or boundaries for media use.	9.1	*13.4* ^a^	<.001	
	My house has rules about what social media profiles are acceptable.	*67.3*	53.02	<.001	
	My house has rules about what privacy settings should be set for social media.	*60.4*	44.9	<.001	
	My house has rules about “friending” someone who is unknown offline.	*67.9*	43.7	<.001	
	My house has rules about “screen-free zones” (rooms or places in the house, such as a bedroom) where no one is allowed to use screens, including televisions, computers, and smartphones.	*25.3*	24.9	.84	
	My house has rules about screen-free times (times when no one is allowed to use media, such as dinnertime) when no one is allowed to use screens, including televisions, computers, and smartphones.	*45.5*	25.9	<.001	
	My house has rules about viewing screens around bedtime.	*43.1*	20.8	<.001	
**Parent social media checking frequency**					<.001
	Once a day or more	78.2	*92.8*		
	A few times a week	*11.5*	4.5		
	Less than once a week	*10.2*	2.7		
**Parent social media posting frequency**					<.001
	Once a day or more	24.6	*61*		
	A few times a week	*31.8*	21.3		
	Less than once a week	*43.6*	17.7		
**Internet time rules: adolescent-reported**					<.001
	Strict internet time rules	54.4	*62.9*		
	Not strict internet time rules	*45.6*	37.1		
**Internet content rules: adolescent-reported**					<.001
	Strict internet content rules	*66.2*	27.2		
	Not strict internet content rules	33.8	*72.8*		
**Communication about internet: adolescent-reported**					<.001
	High-quality communication about the internet	*74.5*	47.6		
	Low-quality communication about the internet	25.5	*52.4*		
**Parent-adolescent relationship: adolescent-reported**					<.001
	More positive parent-adolescent relationship	*77.8*	25.3		
	Less positive parent-adolescent relationship	22.2	*74.7*		

^a^Italicization denotes the class with the majority percentage for each measure.

#### Health Outcomes

Regarding health-related variables, Class 1 participants reported lower levels of physical activity compared with Class 2 participants. However, Class 1 participants also reported *lower* rates of PIU, sleep impairment, depression, anxiety, FOMO, and poor body image compared with Class 2 participants. Table 5 presents these findings.

**Table 5:** Distribution of adolescent health measures included in the latent class analysis in the 2-class model (N=3981).

Variable		Class 1 (n=2501; %)	Class 2 (n=1480; %)	*P* value	
**Physical activity**					<.001
	More physical activity	53.2	*58.9* ^a^		
	Less physical activity	*46.8*	41		
**Daytime sleepiness**					<.001
	Low	*66.9*	16.9		
	High	33.1	*83.1*		
**Depression**					<.001
	No depression	*47.9*	8.2		
	Minimal depression	*40.7*	13.6		
	Mild depression	9.6	*21.9*		
	Moderate depression	1.6	*21.4*		
	Moderately severe depression	0.2	*19.2*		
	Severe depression	0.1	*15.6*		
**Anxiety**					<.001
	Not at risk	*86.6*	30.7		
	At risk	13.4	*69.3*		
**Fear of missing out**					<.001
	Low	*70.5*	14.9		
	High	29.5	*85.2*		
**Body image**					<.001
	High	*70.4*	15.8		
	Low	29.7	*84.2*		

^a^Italicization denotes the class with the majority percentage for each measure.

#### Well-being

Participants in Class 1 scored *higher* on well-being, support, learning, perspective taking, empathetic concern, and communication skills than those in Class 2. Class 1 participants reported less time spent on extracurricular activities compared with Class 2 participants. Table 6 presents these findings.

**Table 6:** Distribution of well-being measures included in the latent class analysis in the 2-class model (N=3981).

Variable		Class 1 (n=2501; %)	Class 2 (n=1480; %)	*P* value	
**Mental well-being**					<.001
	High	*62.9* ^a^	39		
	Low	37	*60.9*		
**Interpersonal reactivity: perspective taking**					<.001
	High	*56.4*	41.4		
	Low	43.6	*58.6*		
**Interpersonal reactivity: empathetic concern**					<.001
	High	*68.9*	23.9		
	Low	31.1	*76*		
**Communication skills**					.001
	High	*52.7*	47.5		
	Low	47.3	*52.6*		
**Comprehensive Inventory of Thriving: support**					<.001
	High	*75.6*	23.2		
	Low	24.4	*76.8*		
**Comprehensive Inventory of Thriving: learning**					<.001
	High	*68.9*	50.2		
	Low	31.1	*49.8*		
**Comprehensive Inventory of Thriving: loneliness**					<.001
	High	*87.1*	32		
	Low	12.9	*69*		
**Extracurricular activities**					<.001
	More participation	*64.3*	44.7		
	Less participation	35.8	*55.3*		

^a^Italicization denotes the class with the majority percentage for each measure.

## Discussion

This study used LCA to develop profiles of media use and parent involvement and their associations with health outcomes and well-being indicators. Although previous studies have illuminated links between media and individual health outcomes [4,89], the LCA classification method provides a rich understanding of the patterns in which adolescents use technology and media as well as an opportunity to understand these patterns alongside the role of parents and health and well-being indicators.

### Previous LCA Findings

Our study findings can first be considered in the context of the few previous studies that have used LCA approaches to study adolescent media use. One previous study focused on quality of life among Swiss adolescents and found 5 distinct classes, with a high social technology use class scoring lowest on moods but highest on social support [90]. Another study focused on technology behaviors, including gaming and internet and smartphone use, among Korean teenagers and assessed psychosocial measures [91]. They found several subtypes, including “dual problem users” who scored highest for addictive technology behaviors and other psychosocial issues. A third study of Australian adolescents found 3 clusters, 1 focused on “instrumental” computer use related to email and general computer use and 2 clusters related to gaming [92]. A fourth study examined physical activity, screen-based media, and self-harm among Chinese adolescents and found that the highest-risk group had low physical activity, high media use, and high self-harm [93]. Our study advances the field by including parents as well as physical health, mental health, and well-being measures. Our study is aligned with previous literature, such as the finding that adolescents who struggle in one domain, such as addictive technology behavior, often have lower health behaviors in other areas, such as psychosocial issues, and concerning health outcomes, such as sleep impairment [93].

### Study Findings in the Context of Previous Literature

Previous studies exploring the potential of media use to negatively affect adolescents have produced mixed results, leading some to argue that these effects are present for *some* adolescents but not others. Research suggests that the effects of social media on adolescent well-being vary so widely that media use has positive effects on some adolescents and negative effects on others [94]. This finding has prompted a call for research to consider a differential susceptibility model to identify those individual differences that are likely to have susceptibility to negative impacts [95].

Our study builds upon and expands on these previous findings in several ways. First, our study includes both negative and positive impacts, here defined as risk and health outcomes, using previous evidence to define these categories. Aligned with the differential susceptibility model, we found a 2-class model illustrating that most adolescents who use media do well across health behaviors and outcomes. A smaller proportion of adolescents are at higher risk, and this risk extends across their technology behaviors as well as health behaviors. A critical difference between Class 1 and Class 2 was their reported level of sleepiness, with Class 2 participants reporting far greater sleep impairment compared with Class 1 participants. Given the substantial impact that sleep can have on both mental and physical health [96,97], this finding bears further exploration and could be considered a target of future interventions to reduce negative health consequences.

Second, our study included parents as a key construct in understanding the links between technology use and health outcomes. Parents are also the gatekeepers of device acquisition and ownership. We assessed the role of parents as moderators, supporters, and role models in the relationship between adolescent technology use and health and well-being outcomes. Compared with Class 2 participants, we found that Class 1 participants were more likely to have family-owned devices than personally owned devices. Furthermore, adolescents in Class 1 were more likely to have household rules that were often centered on content, coviewing, and communication. Class 2 parents were more likely to report no rules, although Class 2 adolescents were more likely to report strict rules around screen time. These mixed findings may suggest that, for some families, the rules are unclear or are stated but not reinforced. Parents in Class 2 were also more likely to report high levels of their own daily social media use. Finally, Class 1 adolescents were more likely to report positive parent-child communication about technology use and a more positive parent-child relationship in general compared with Class 2 adolescents. The consistency and conceptual connection between these variables is a critical finding of our study and a significant advancement in how we understand the balance of health and risk from adolescents’ technology use. This study’s findings support the positive role that engaged parents can play in promoting health and preventing harm among adolescents related to technology use. Thus, we propose to name the larger Class 1 *Family-Engaged Adolescents* and the smaller Class 2 *At-Risk Adolescents*.

### Study Findings in the Context of Emerging Paradigms

When seeking to frame the role of technology in our health and well-being, one often hears terms such as *online and offline* or *online and in-real-life*. These terms promote a separation of our daily lives into 2 distinct worlds. News stories often frame technology as something that should be reduced or avoided, which could influence a view of technology as a risk behavior itself. However, today’s teenagers have digital tools woven into the fabric of their everyday existence. For adolescents, technology may be seen as within and not separate from their world and as something that can lead to healthy and less healthy outcomes and experiences. To go beyond this dichotomous view of technology, many researchers, policy makers, and families seek new frameworks to consider and describe the role of technology in how we navigate today’s world. Our study supports this more comprehensive viewpoint as the findings illustrate the strong alignment within each of our 2 classes across physical health behaviors, technology behaviors, parenting engagement, and mental and well-being outcomes.

An emerging framework that aligns with our study findings is the Human Experience framework (HX) [98]. The HX approach seeks to define technology as part of the human experience. As such, it can be associated with both everyday and special interactions and with both positive and negative experiences. Applying HX to our approach to youth technology use may allow us to avoid oversimplified categorizations of technology use as *bad* or a *risk behavior* and see technology use as a multifaceted activity that may be used in healthy and unhealthy ways. Thus, the HX approach may be a step toward a more comprehensive approach to understanding the role of technology in young people’s lives and their differential susceptibility to its benefits and risks based on their lived experience. Furthermore, the HX approach may be a useful lens for technology developers to see their products as part of a larger set of life experiences that may affect or influence the adolescent developmental period.

### Limitations

The first limitation of this study is that our results may not generalize beyond the study population of early adolescents recruited via Qualtrics. Recruiting from a web-based panel of participants meant that we could designate the study population size and criteria but limited our ability to assess the external validity of the sample. However, the Qualtrics platform and panels have been used in other studies of early adolescents [58], and the panels have been found to have close approximations to the US population [56]. Second, LCA provides a systematic approach to creating profiles representing critical variables, although our interpretations of those profile groupings may have inaccuracies. Furthermore, other unmeasured variables such as other family or school factors may have influenced our study outcomes. This study used cross-sectional data, which is common within the LCA approach but does not allow for an understanding of long-term predictors or consequences. All measures were self-reported, and future studies including other novel measurement approaches, such as biological or cognitive studies, are needed.

Finally, it may be notable to readers that we did not investigate any specific platforms in this study. Rather, we focused on technology devices and social media use frequency. Furthermore, we incorporated the technology use importance scale (ADTI), which assesses critical interactions and functions of technology that may apply to many different platforms. This was a purposeful approach toward better understanding the mechanisms underlying technology use rather than the role of specific platforms.

### Implications and Future Directions

This study raises important implications for how we approach the topic of adolescent technology use in health care, policy, and research. Too often, the dialogue around adolescent technology use has been to frame it as a negative risk behavior that all adolescents should cease [99]. Our findings support that, for a smaller group of adolescents in this study who made up *At-Risk Adolescents* (Class 2), their higher rates of technology ownership and use were associated with higher rates of health risks and lower scores on well-being indicators. However, our findings suggest that two-thirds of the adolescents who made up *Family-Engaged Adolescents* (Class 1) in our study integrated technology into their lives in ways that were not associated with higher rates of depression, anxiety, or other poor health outcomes. Thus, the study findings indicate that most adolescents using technology do so in ways that do not lead to increased risk of negative health consequences.

We propose that a critical factor that affected *Family-Engaged Adolescents*’ (Class 1) health and well-being was the role of parents. *Family-Engaged Adolescents* were more likely to have household media rules at home regarding media and technology use. Participants in this group were more likely to report that the rules they had at home were aligned with the recommendations of the AAP on content, communication, and coviewing. *Family-Engaged Adolescents* were more likely to communicate with their parents about their technology use and, overall, they reported a positive relationship with their parents compared with the *At-Risk Adolescents* (Class 2). Furthermore, Class 1 parents used social media less frequently compared with Class 2 parents, highlighting the integral aspect of role modeling that parents have regarding technology use.

There are several concrete recommendations that this study supports. First, consistent with AAP recommendations, we recommend a shift away from rules centered on screen time. Our evidence supports that household rules focused on content, communication, and coviewing were more likely to be associated with lower health risk and improved well-being (see Multimedia Appendix 1 for more information). The findings of this study can direct pediatricians’ and other health care providers’ counseling toward parents and encourage them to leverage these approaches at home. Providers can also partner with parents to ensure that messaging around media is culturally relevant and developmentally appropriate. Health care providers may benefit from using technology within the clinic visit to share these recommendations, such as through electronic health record prompts to ask about technology behaviors and home rules and after-visit summary resources, including recommendations and links to resources.

These resources may include tools that can support parents in creating media rules at home. First, the Family Media Use Plan of the AAP has content, communication, and coviewing as key elements, and this approach is without cost. Other for-profit web-based tools such as Circle and Bark may integrate content restrictions into family rule development, although these programs are also at a cost and often focus predominantly on screen time. Including adolescents in the discussion and selection of rules is a critical tactic to obtaining their buy-in for setting limits and boundaries.

Second, health care providers and researchers should consider the integral role that parents play in their children’s media use. In addition to recommending that parents create and enforce household media rules, pediatricians can support parents in developing positive relationships with their adolescents. These approaches may include coviewing media. Counseling parents about having awareness of their own technology use and their role modeling of technology behaviors may be a critical recommendation to influence health outcomes. Researchers designing interventions must consider the role of parent support, both related to technology and likely more broadly in adolescents’ lives, if they want to truly affect adolescent well-being. Finally, we encourage future research and policy to consider technology as integrated into adolescents’ daily lives. Our study supports the exploration of new frameworks such as the HX approach toward the design of new policies and studies to advance adolescent health (see Multimedia Appendix 2 for more information).

## Appendix

Multimedia Appendix 1 Parent tip sheet.

Multimedia Appendix 2 Tech tip sheet.

## Abbreviations

AAP: American Academy of Pediatrics
ADTI: Adolescents’ Digital Technology Interactions and Importance
CIT: Comprehensive Inventory of Thriving
FOMO: fear of missing out
HX: Human Experience framework
LCA: latent class analysis
PHQ-9: 9-item Patient Health Questionnaire
PIU: problematic internet use
VR: virtual reality
