# Supplementary material for: Digital Technology and Media Use by Adolescents: Latent Class Analysis
Source: JMIR Pediatr Parent. 2022 May 4;5(2):e35540. doi: 10.2196/35540 (PMC9118083; doi:10.2196/35540)
Supplement: Multimedia Appendix 1 [file pediatrics_v5i2e35540_app1.pdf]

SMAHRT
SOCIAL MEDIA & ADOLESCENT HEALTH RESEARCH TEAM

CENTER FOR SCHOLARS & STORYTELLERS

How **parents** can support the **well-being of youth** around digital technology and media

Our research study:

4,000 adolescents (13 -18) and their parents were surveyed.

Questions covered technology ownership and use, parental involvement, health outcomes, and well-being indicators.

A latent class analysis was used to sort participants into groups or classes.

Results found two classes: **Family-Engaged** and **At-Risk** adolescents.

"We know that many past research studies have focused on large groups of youth. These studies have found very small overall effects of technology on adolescents. In our new study, we wanted to better understand whether there were unique groups within the adolescent population." (Dr. Megan Moreno, lead author)

Class 1: "Family-Engaged Adolescents"

- Technology devices were owned by their families.
- Have rules at home about technology use, focused on content and they communicate about them frequently.
- Reported higher levels of well-being, sleep, and physical activity.
- Reported high levels of positive, ongoing communication and relationships with parents.
- Parents' own social media use was low.

Class 2: "At Risk Adolescents"

- Technology devices were owned by the teen.
- There were either no rules at home about technology use, or strict rules about screen time.
- Reported higher levels of risk such as depression, anxiety, loneliness, and poor body image.
- Reported low levels of communication with their parents about rules or otherwise.
- Parents' own social media use was high.

May 2022

SMAHRT
SOCIAL MEDIA & ADOLESCENT HEALTH RESEARCH TEAM

CENTER FOR SCHOLARS & STORYTELLERS

Based on study results, we recommend:

1. **Consider family-owned technology devices, rather than individual technology devices.**

Why? Family-owned devices may support communication about technology or co-viewing.

---

2. Create and maintain household rules centered on content and communication.

Why? Rules focused on content and ongoing parent communication could lead to improved well-being for adolescents.

---

3. Parents should be aware of their own technology use, particularly at home and when using social media.

Why? Parent social media use may impact parent-adolescent communication and well-being outcomes for their adolescents.

---

4. Parents can play a role in promoting healthy technology use and well-being behaviors and should start when their children are younger.

Why? The **Family-Engaged** group had higher well-being outcomes than the **At-Risk** group.

About the Social Media and Adolescent Health Research Team (SMAHRT):

The Social Media and Adolescent Health Research Team (SMAHRT), based at the University of Wisconsin – Madison, strives to advance society's understanding of the relationship between media and adolescent health.

For more information contact: smahrt@pediatrics.wisc.edu

About The Center for Scholars & Storytellers:

The Center for Scholars & Storytellers, based at UCLA, bridges the gap between researchers and content creators to support positive youth development.

For more information contact: info@scholarsandstorytellers.com

Additional Sources:

Moreno MA, Binger K, Zhao Q, Eickhoff J, Minich M, Uhls, YT. Adolescent digital technology and media use: A latent class analysis. JMIR Pediatrics and Parenting. 2022.

American Academy of Pediatrics. (2015). Kids & Tech: 10 Tips for Parents in the Digital Age.

HXproject.org. (2022, February 2). Home. HX : It's Our HUMAN EXPERIENCE. https://hxproject.org/

Moreno, M. A., Binger, K., Minich, M., Zhao, Q., & Eickhoff, J. (2022). Adolescent digital technology interactions and importance: associations with depression and well-being. Cyberpsychology, Behavior, and Social Networking.

Protecting Youth Mental Health: The U.S. Surgeon General's Advisory. Office of the Surgeon General. Published online December 6, 2021.

Uhls, Y. T. (2016). Media moms & digital dads: A fact-not-fear approach to parenting in the digital age. Routledge.
