# Supplementary material for: Digital Technology and Media Use by Adolescents: Latent Class Analysis
Source: JMIR Pediatr Parent. 2022 May 4;5(2):e35540. doi: 10.2196/35540 (PMC9118083; doi:10.2196/35540)
Supplement: Multimedia Appendix 2 [file pediatrics_v5i2e35540_app2.pdf]

Four Evidence-Based Calls to Action for the **Tech Industry**

To support the digital literacy and well-being of youth from all backgrounds

The study:

4,000 adolescents (13 - 18) and their parents were surveyed.

Questions covered technology ownership and use, parental involvement, health outcomes, and well-being indicators.

A latent class analysis was used to sort participants into two classes: **Family-Engaged** and **At Risk** adolescents

"The issue of technology and our kids is something that is on our minds every single day, multiple times a day. How to regulate, how to put boundaries, and how to help them so they're not completely overwhelmed by the amount of information." -Parent (HXproject.org, 2022)

Class 1: "Family-Engaged Adolescents"

- Technology devices were owned by their families.
- Have rules at home about technology use, focused on content and communicate about them frequently.
- Reported higher levels of well-being, sleep, and physical activity.
- Reported high levels of positive, ongoing communication and relationships with parents.
- Parents' own social media use was low.

Class 2: "At Risk Adolescents"

- Technology devices were owned by the teen.
- There were either no rules at home about technology use, or strict rules about screen time.
- Reported higher levels of risk such as depression, anxiety, loneliness, and poor body image.
- Reported low levels of communication with their parents about rules or otherwise.
- Parents' own social media use was high.

SMAHRT
SOCIAL MEDIA & ADOLESCENT HEALTH RESEARCH TEAM

CENTER FOR SCHOLARS & STORYTELLERS

1. Incorporate wellness checks and content guidelines directly into the technology platforms adolescents engage with.

Why? 87% of tweens and teens have gone online for mental health information (HXproject.org, 2022).

At risk adolescents reported higher levels of risks such as depression, anxiety, loneliness and poor body image.

2. Provide youth more agency and flexibility in accessing the content they want to see, rather than prioritizing content by popularity or profit margin.

Why? Kids that thrive (i.e. **Family-engaged adolescents**) are more likely to have household technology rules that emphasize content rather than time limits.

92% of teens looked for information on mental health topics after watching a popular TV show (Levinson, et. al., 2021).

3. Educate technology developers with research-informed training on the range of needs of the youth that will use their products.

Why? "When not deployed responsibly and safely, these tools can pit us against each other, reinforce negative behaviors like bullying and exclusion, and undermine the safe and supportive environments young people need and deserve." (Protecting Youth Mental Health: The U.S. Surgeon General's Advisory 2021).

There is a significant difference between **at-risk adolescents** and **family-engaged adolescents** in patterns of behavior within the constructs of technology ownership and use, parental involvement, health outcomes and well-being indicators.

4. Support digital parenting by reworking parental controls to focus less on restrictions and more on fostering communication and connection between parent and child on technology platforms.

Why? Key factors that were associated with better health and well-being for adolescents included household rules about content, active parental media involvement, less parental posting and checking of their own social media, and active conversations with their parents about technology.

The highest risk of problematic internet usage occurred when parents were perceived as unsupportive, even when adolescents' mental well-being was high (Hwang & Toma, 2021).

“Media is always an easy target. Instead, we should be examining the bigger issues that are affecting young people — inequality, poverty, climate, education. We need to be looking at the systems that young people are growing up in. Social media is only one part of it.” -Dr. Yalda T. Uhls (EdSource, 2022)

About the Social Media and Adolescent Health Research Team (SMAHRT):

The Social Media and Adolescent Health Research Team (SMAHRT), based at the University of Wisconsin – Madison, strives to advance society’s understanding of the relationship between media and adolescent health.

For more information contact: smahrt@pediatrics.wisc.edu

About The Center for Scholars & Storytellers:

The Center for Scholars & Storytellers, based at UCLA, bridges the gap between researchers and content creators to support positive youth development.

For more information contact: info@scholarsandstorytellers.com

Additional Sources:

Hwang, J., & Toma, C. L. (2021). The Role of Mental Well-Being and Perceived Parental Supportiveness in Adolescents’ Problematic Internet Use: Moderation Analysis. JMIR Mental Health, 8(9), e26203. https://doi.org/10.2196/26203

HXproject.org. (2022, February 2). Home. HX : It’s Our HUMAN EXPERIENCE. https://hxproject.org/

Levinson, J. A., Tsai, E. M., Felt, L., Wartella, E., & Uhls, Y. T. (2021, May). Seeking Support: Evaluating the Impact of 13 Reasons Why on Adolescent Mental Health. Center for Scholars & Storytellers. https://drive.google.com/file/d/1LsrLFOVNvfrqm1zFGt5OvrJ_e7BEz7SF/view

Moreno MA, Binger K, Zhao Q, Eickhoff J, Minich M, Uhls, YT. Adolescent digital technology and media use: A latent class analysis. JMIR Pediatrics and Parenting. 2022.

Protecting Youth Mental Health: The U.S. Surgeon General’s Advisory. Office of the Surgeon General. Published online December 6, 2021.
